# Scarred Lung. An Update on Radiation-Induced Pulmonary Fibrosis

**DOI:** 10.3389/fmed.2020.585756

**Published:** 2021-01-15

**Authors:** Natalia Jarzebska, Ekaterina S. Karetnikova, Alexander G. Markov, Michael Kasper, Roman N. Rodionov, Peter M. Spieth

**Affiliations:** ^1^Department of Anesthesiology and Critical Care Medicine, University Hospital Dresden, Technische Universität Dresden, Dresden, Germany; ^2^Division of Angiology, Department of Internal Medicine III, University Center for Vascular Medicine, University Hospital Dresden, Technische Universität Dresden, Dresden, Germany; ^3^Department of General Physiology, Saint-Petersburg State University, Saint Petersburg, Russia; ^4^Institute of Anatomy, Technische Universität Dresden, Dresden, Germany

**Keywords:** radiation, pulmonary fibrosis, macromolecular damage, nitric oxide, dimethylarginine dimethylaminohydrolase

## Abstract

Radiation-induced pulmonary fibrosis is a common severe long-time complication of radiation therapy for tumors of the thorax. Current therapeutic options used in the clinic include only supportive managements strategies, such as anti-inflammatory treatment using steroids, their efficacy, however, is far from being satisfactory. Recent studies have demonstrated that the development of lung fibrosis is a dynamic and complex process, involving the release of reactive oxygen species, activation of Toll-like receptors, recruitment of inflammatory cells, excessive production of nitric oxide and production of collagen by activated myofibroblasts. In this review we summarized the current state of knowledge on the pathophysiological processes leading to the development of lung fibrosis and we also discussed the possible treatment options.

## Introduction

Radiation therapy is an integral part of the treatment of various malignant neoplasms (1), including tumors of the thorax (2–4). Radiation induced pulmonary fibrosis is a common complication of this therapy affecting 5–50% of patients, which significantly limits available treatment options even after successful eradication of the tumor itself (5). Radiation-induced lung injury can be divided into two distinct forms: (1) classical radiation pneumonitis, which is restricted to the irradiated area and leads to fibrosis and (2) sporadic radiation pneumonitis, affecting much broader area than the tissue which was subjected to irradiation and resulting in bilateral lymphocytic alveolitis, mediated by various immunological processes (6, 7). Currently, corticosteroids, azathioprine, and cyclosporin A are used as first line treatment for post-radiation pneumonitis (8, 9). Ambroxol and angiotensin-converting enzyme inhibitors are suggested as additional drugs to prevent or attenuate the extent of pneumonitis (10, 11). Lung fibrosis, however, is an end stage of radiation-induced pneumonitis and in most of the cases it is resistant to all currently known pharmacological interventions. Previously only symptomatic treatment with antitussive agents, oxygen therapy, and mechanical ventilation was available (9). More recently, it was shown that nintedanib, a multiple tyrosine kinase inhibitor, and pirfenidone, a TGF-β inhibitor, exhibit anti-inflammatory and anti-fibrotic effects in experimental murine irradiation models (12, 13) and these drugs are now in clinical use for treatment of pulmonary fibrosis (13, 14).

The aim of this review is to summarize and analyse the currently available data on the pathophysiological mechanisms leading to lung fibrosis. Hopefully it will allow identifying promising directions for further research, which will lead to the development of effective preventive and/or treatment approaches to tackle post-radiation pulmonary fibrosis.

## Clinical Perspective

The lung parenchyma is one of the most radiation-sensitive tissues with the alveolar-capillary unit being the most vulnerable part of the lung (15). The likelihood of developing side effects of radiation therapy, like difficulty swallowing, shortness of breath, breast or nipple soreness and shoulder stiffness depends on many factors like patient-related variables, the nature of the tumor itself and the parameters of radiation therapy (16–18). Specifically, it has been demonstrated that age above 65 years, smoking, presence of co-morbidities and poor pulmonary function tests (decreased forced expiratory volume and poor diffusing capacity of the lungs for carbon monoxide) have been associated with increased incidence of radiation pneumonitis (19, 20). Additionally, radiation therapy administered for mid and lower lobe lung tumors, breast cancer with tangential fields and esophageal cancer are also correlated with significantly increased incidence of radiation pneumonitis (20). Other risk factors include more than 30% of the planned target volume receiving 20 Gy or more, and the use of certain medications like bleomycin, cyclophosphamide, vincristine, taxanes, doxorubicin, dactiomycin, mitomycin, gemcitabine, erlotinib and bevacizumab (21–25). There are also certain molecular markers reported, which could be used to improve individualized treatment. For example, patients with elevated post treatment serum TGF-β1 (transforming growth factor beta 1) levels above baseline exhibit a significantly higher risk of radiation induced lung injury (26, 27). Furthermore, persistently elevated IL1a (interleukin 1 alpha) and IL6 (interleukin 6), ICAM 1 (intercellular adhesion molecule 1), SP-A (surfactant protein A) and SP-D (surfactant protein D) serum levels are predictive for the development of radiation pneumonitis (28–30). Furthermore, certain ATM (ataxia telangiectasia mutated) gene polymorphisms have been associated with increased risk of radiation pneumonitis (31). Finally, there are also studies showing that the risk of severe radiation pneumonitis is increased in patients with pre-existing idiopathic pulmonary fibrosis (IPF) (32–34). Nonetheless, there are no formal guidelines limiting the eligibility of IPF patients for thoracic radiotherapy, even though according to the recommendations of the European Organization for Research and Treatment of Cancer radiotherapy for lung cancer should be avoided in IPF patients (35). The decision of whether or not to treat these vulnerable patients with irradiation of the thorax should be made after a careful evaluation of a multidisciplinary team taking into account a comprehensive risk assessment. Clearly more research is needed to establish the safest treatment options. The group of Yoshitake and colleagues investigated the side effects of thoracic stereotactic body radiotherapy (SBRT) in patients with interstitial lung changes (ILC) and found that the risk of radiation pneumonitis grade 2 or more was increased in patients with subclinical ILC (36). Even though the topic still requires more research, dose limits for SBRT in patients with pre-existing ILC are recommended (37). There is an increasing body of evidence suggesting that proton beam therapy could be a safer option in patients with IPF than conventional radiotherapy or SBRT (38–41). However, prospective, multicentre studies with large number of patients enrolled are still needed. Nintedanib is a relatively new drug that can be used to slow the progression of IPF. It is a tyrosine kinase inhibitor affecting tyrosine phosphorylation on platelet-derived growth factor, vascular endothelial growth factor and fibroblast growth factor leading to suppression of inflammation, angiogenesis and fibroblast activation (42). The drug has been shown to prevent/retard acute exacerbations in patients with IPF (43, 44), however it was not effective in alleviating bleomycin-induced pulmonary fibrosis in an animal model (45).

Apart from causing pneumonitis and pulmonary fibrosis, radiotherapy is also thought to contribute to activation of cancer-associated fibroblasts (CAF), at least in some conditions. CAFs are a heterogeneous group of stromal cells inside a tumor, which differ epigenetically and phenotypically from normal fibroblasts. The major cellular origin of CAFs are normal fibroblasts that are transformed by the tumor microenvironment, however, smooth muscle cells, pericytes, adipocytes, mesenchymal stem cells and endothelial cells are also demonstrated to be the source of CAFs (46, 47). Those cells modulate the composition of the extracellular matrix through secretion of growth factors and cytokines which lead to regulation of tumor proliferation, invasion and the potential for metastasis. Interestingly, there are also studies demonstrating that CAFs can slow down tumor progression by formation of a physical barrier which limits tumor growth and the possibility to migrate (48). Radiotherapy affects the proliferation of CAFs at the genetic level, but does not limit the ability of these cells to sustain a microenvironment which supports tumor growth (49). The influence of radiotherapy on the CAFs is a matter of ongoing investigations. On one hand, there is data supporting the idea that irradiation can modify the CAFs leading to abrogation of tumor promoting ability (50), however, there are also studies showing that irradiated fibroblasts can induce epithelial-to-mesenchymal transition (EMT) in cancer cells and promote invasiveness through elevation of TGF-β levels (51, 52). In line with this data, the group of Ohuchida et al. showed that irradiated CAFs increase the invasiveness in pancreatic cancer cells (53). In another study it was demonstrated in the same type of cancer that CAFs after irradiation induce EMT and invasiveness by activation of the P38 pathway (54).

## Cascade of Pathophysiological Processes Leading to Lung Fibrosis

### Primary and Secondary Damage to Macromolecules

Exposure to radiation causes both direct and indirect macromolecular damage and also triggers the generation of various reactive oxygen species (ROS), including superoxide (O_2_−), hydrogen peroxide (H_2_O_2_) and hydroxyl radical (⦁OH) (55, 56). The combination of irradiation itself and the generation of ROS affect all types of macromolecules, including DNA, proteins, proteoglycans and lipids. However, double-strand breaks in DNA have most severe consequences for the cells (57). Primary and secondary damage triggers cascades and networks of biochemical reactions and the balance between them determines if normal lung tissue will be restored or if the fibrotic response will be initiated (58).

### DAMPs-TLRs-Pro-Inflammatory Cytokines

Cell damage caused by irradiation leads to the accumulation of damage-associated molecular patterns (DAMPs) in the intercellular space of the lungs. The most prevalent types of DAMPs include extracellular DNA, extracellular ATP, high mobility group box chromosomal protein B1, heat-shock protein 70, uric acid and low-molecular hyaluronan (59). These DAMPs are generated in aseptic conditions and activate cell surface-bound TOLL-like receptors (TLR) 2 and 4 (18). TLRs are expressed in many cells, including alveolar epithelial cells of type II (60), endothelial cells, alveolar macrophages, fibroblasts, dendritic cells, monocytes, lymphocytes, neutrophils and natural killer cells (18, 61, 62) and their activation triggers sequential release of different mediators, including pro-IL-1β, pro-IL-18 and type I interferon (63). Interestingly, studies on the development of post-radiation pulmonary fibrosis in mice with global deficiency of TLR2 and TLR4, as well as with the knockout of MyD88 (Innate Immune Signal Transduction Adaptor) showed that these mice developed more severe pulmonary fibrosis compared to wild-type mice (60, 64, 65). This fact indirectly indicates that these signaling cascades promote regeneration of lung tissue rather than damage.

### Pyroptosis Pathways of Inflammasome Activation

Simultaneously with the stimulation of synthesis of pro-IL1β, pro-IL18, and type I interferons, irradiation causes pyroptosis, a highly inflammatory form of programmed cell death (66). Specifically, irradiation can activate non-active multi-protein signaling complexes NLRP3 (NLR family, pyrin domain-containing 3) present in inflammasomes of various lung cells (18, 67, 68). The activation can occur directly through ROS (69) or indirectly through DAMPs like ATP, ADP, and adenosine which bind to P2XR, P2YR, and P1R (ATP-gated ion channels) (70) and through low molecular weight hyaluronan activating the surface receptors CD44 (69). Another possible pathway of activation is through the outflow of cell content, particularly uric acid, caused by irradiation-mediated damage to cell membranes (18, 68). Activated inflammasomes initiate activation of caspase-1 which cleaves pro-IL-1β and pro-IL-18 with the formation of active IL-1β and IL-18 (71–73) and also disrupts the integrity of the outer cytoplasmic membranes, which leads to osmotic lysis of cells and increases the amount of DAMPs in the intercellular milieu (73, 74). These processes lead to increased synthesis of proinflammatory cytokines and pyroptosis in lung tissue. IL-1β also plays an important role in initiation of the acute inflammatory responses by binding to its ubiquitously expressed receptor (IL-1R-1) and stimulation of the production of TGF-β, pro-IL-1β, TNF-α, and IL- 6. Together these cytokines promote the recruitment and activation of innate immune cells, as well as trigger and enhance cascades of aseptic inflammation in the damaged part of the lung (18, 71).

### P2X Purinoreceptor 7

The NLRP3 inflammasome might be also involved in the pathogenesis of radiation-induced fibrosis by activating purinergic P2X and P2Y receptors (purinergic signaling). Purinergic signaling is involved in ionizing radiation-induced biological effects (75). An important protein in these pathways is the ATP-stimulated P2X purinoreceptor 7 (P2X7R), selectively present in alveolar macrophages and in alveolar epithelial type I cells in lungs and known to regulate the activation of the NLRP3 inflammasome. A rapid increase in the extracellular ATP concentration, for example after damage of a tissue or after cell death, causes an endogenous signal of danger and activates the NLRP3 inflammasome by binding of ATP to P2X7R which functions as ligand-controlled ion channel (76). The effect of ATP may be transmitted by P2X7R, which causes the formation of pannexin-1 pores. Their opening leads to a fast outflow of K^+^-ions from the cytosol (77). The decrease in the cytoplasmic K^+^ concentration causes assembly and activation of the inflammasome leading to an auto- catalytic cleavage of the inactive procaspase-1 to its active form. The inflammasome transmitted caspase-1-dependent proteolytic cleavage of inactive proforms of cytokines of the IL-1-family (pro-IL-1β, pro-IL-18) results in biological active forms, which are released from cells as a part of the inflammatory reaction. Further, the involvement of the connexin43 hemichannel in the ATP release downstream of the P2X7R in response to irradiation has to be taken into account (78). An upregulation of connexin43 in alveolar epithelial cells of rats with radiation-induced pulmonary fibrosis has been described earlier (79). Furthermore, P2X7R knockout mice exhibited dramatically reduced lung inflammation and fibrosis, underlining the important role of P2X7R in fibrotic diseases (80).

### Inducible Nitric Oxide Synthase (iNOS), Asymmetric Dimethyarginine (ADMA) and Dimethyarginine Dimethyaminohydrolase (DDAH)

IL-1β, TNF-α, and other cytokines activate iNOS, which starts to produce large quantities of NO. When nitric oxide is produced, it can bind to O_2_− with the formation of peroxynitrite (ONOO^−^) causing secondary damage to macromolecules (DNA, proteins, glucosaminoglycan hyaluronan, etc.) (81). The importance of peroxynitrite in lung fibrosis development has been demonstrated in animal studies where is was shown that iNOS knockout mice had significantly less fibrotic changes in the lungs compared to wild-type mice after bleomycin treatment (81–83). The activity of iNOS *in vivo* is regulated by asymmetric dimethylarginine (ADMA), its endogenous inhibitor. It has been shown that at concentrations exceeding 10μM ADMA not only decreases NO production by iNOS but also uncouples it with the production of O_2_− (84). Therefore, it was speculated that the addition of ADMA to already fully active iNOS cannot reduce the damage to the lung tissue mediated by NO, but paradoxically can lead to further exacerbation of the damage due to the peroxynitrite formation. This assumption was confirmed by Wells and colleagues in *in vitro* and *in vivo* studies (84). They showed that ADMA elevates collagen production in primary mouse lung fibroblasts and that ADMA infusion via osmotic minipumps for 2 weeks caused collagen deposition in mice lungs (85). In the same manuscript the authors proposed an interesting pathway of pro-fibrotic ADMA activity where ADMA increases arginase activity leading to elevated levels of ornithine and urea; ornithine is a precursor of proline, an amino acid essential for collagen synthesis (85). In line with these observations, other study demonstrated that 21 days after bleomycin injury there was no difference in the fibrotic response in mice supplemented with ADMA and those given placebo (83). Concentration of ADMA is regulated by the activity of dimethylarginine dimethylaminohydrolase (DDAH), an enzyme which cleavages ADMA (86, 87). DDAH expression and activity increase in course of lung fibrosis development, through TGF-β and IL- 6 which increase mRNA levels of DDAH2 (one of two DDAH isoforms) (83); and through IL-1β which enhances DDAH activity and rises its intracellular concentrations (88). Increased DDAH expression and activity in turn reduces ADMA concentration in lung tissue, which results in greater amounts of NO produced by iNOS, contributing to further damage of lung tissue.

### Sources of Fibroblasts: Epithelial-Mesenchymal Transition (EMT), Endothelial-Mesenchymal Transition (EndMT) and Recruitment of Fibrocytes and Myofibroblasts

Increased concentrations of TGF-β1 in course of development of lung fibrosis induce EMT (epithelial-mesenchymal transition) of alveolar epithelial cells type II (AET II cells) (89) and EndMT (endothelial-mesenchymal transition) of microvascular endothelial cells (90). During EMT and EndMT, the polarized epithelial and endothelial cells loose their polarity and specific markers such as E- or VE-cadherin, tight junction proteins etc., increase expression of mesenchymal markers (vimentin, collagens I and III, α-SMA etc), move to the interstitium and gain phenotype like mesenchymal cells (89). A convincing proof of EMT *in vivo* was demonstrated in lineage-tracing studies (91–93) where mice with selective expression of β-galactosidase (β-gal) only in lung epithelial cells were used. After induction of lung fibrosis by TGF-β1 (92), thorax irradiation (91) or by bleomycin treatment (93) the authors reported the appearance of expression of different mesenchymal markers (α-SMA, vimentin, S100A4) in β-gal-positive cells and the co-expression of mesenchymal markers and pro-surfactant C (a marker of AET II cells) (91–94). There is a controversy about the contribution of epithelial cells to myofibroblast population and to the physiological role of EMT *in vivo* (95, 96). Moreover, recent findings suggest that AET II cells undergoing EMT promote a pro-fibrotic microenvironment through paracrine signaling activating local fibroblasts (97). An EMT of AET I cells is unknown. The various cytokines produced during aseptic inflammation also promote the recruitment of fibrocytes from the peripheral blood and their differentiation into fibroblasts in the lung tissue, where they produce extracellular matrix (93, 98). The last step in the development of radiation-induced pulmonary fibrosis is the formation of myofibroblasts from fibroblasts (99). The cellular source of myofibroblasts has been a subject of debate in recent years. It was suggested that the myofibroblasts pool is heterogeneous and derives from multiple sources, such as resident fibroblasts, circulating fibroblasts, perivascular mesenchymal cells, and alveolar epithelial cells (95, 100–102). However, recent studies using genetic lineage tracing identified the resident lipid-droplet-containing interstitial fibroblasts, as a precursor cell for the myofibroblast, at least in the bleomycin model of lung fibrosis in mice (103). Regardless of the source, the differentiation of fibroblast into myofibroblasts is driven by TGF-β1 (103). Upon activation these cells produce excessive amounts of extracellular matrix (ECM) (fibronectin, collagen etc), which causes contraction of the lung tissue (104, 105) and increase the thickness of the alveolar-capillary membrane together leading to irreversible compromised diffusing capacity of the lung (106) and restrictive ventilatory insufficiency.

**Figure 1:**
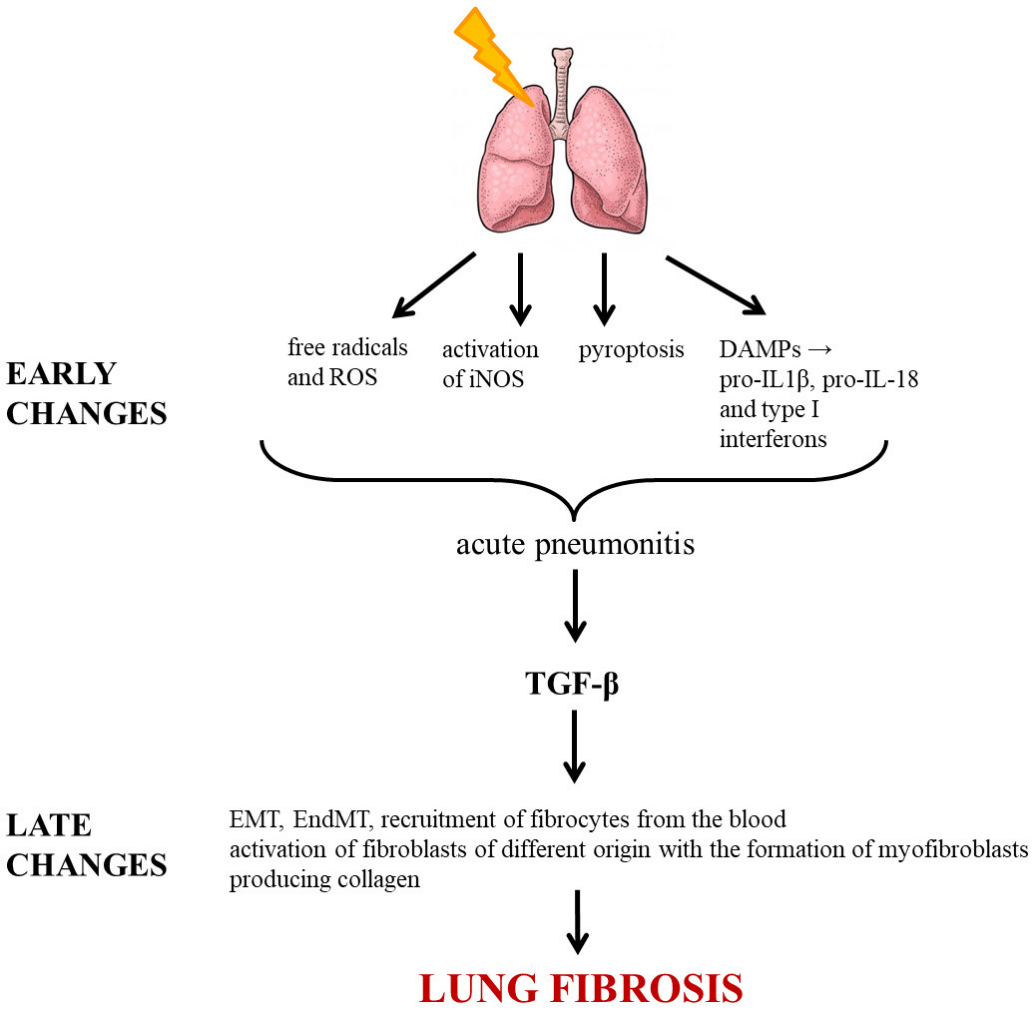
Molecular pathways triggered by irradiation leading to lung fibrosis. ROS, reactive oxygen species; DAMP, damage-associated molecular pattern; IL, interleukin; iNOS, inducible nitric oxide synthase; TGF-β, transforming growth factor beta; EMT, epithelial-mesenchymal transition; EndMT, endothelial-mesenchymal transition.

The various processes leading to irradiation-induced lung fibrosis are summarized in Figure 1.

## Research Targets Regarding Lung Fibrosis Prevention and Treatment

### Minimizing Protein Damage

Since the primary and secondary damage to macromolecules is the starting point for the development of all pathophysiological mechanisms of lung fibrosis, one of the therapeutic approaches considered was the use of a non-toxic anti-ulcer drug geranylgeranlyacetone (GGA), which induces the expression of intracellular chaperone heat shock protein 70. The plausibility of this approach was shown by the group of Kim and colleagues in a study where they treated mice with GGA before and after irradiation and found out that after 6 months the animals had less pronounced lung fibrosis and had less signs of EMT in lung tissue compared with the placebo group (107). However, one possible drawback in using this drug in clinical practice is that it can also reduce the efficacy of irradiation as an anti-tumor treatment (108).

### Influence on DDAH-ADMA-iNOS-NO Axis

The second possible therapeutic approach is based on the rationale that activated iNOS in the development of lung fibrosis is the source of excess of nitric oxide, which causes secondary injury to the lung. Studies in mice showed that at least in the bleomycin model the use of iNOS inhibitors resulted in significant reduction in the degree of lung fibrosis compared to animals from the placebo groups (81, 83). Pullamsetti and colleagues demonstrated a protective role of a selective DDAH inhibitor L-291 in the development of bleomycin-induced lung fibrosis. Interestingly, the inhibitor seemed to affect both ADMA-independent (antifibrotic) and ADMA-dependent (antiproliferative) pathways of lung fibrosis development (83).

### Reducing TGF-β1-Mediated Effects

According to the current state of knowledge, TGF-β1 plays a key role in the development of lung fibrosis (108). Therefore, many efforts are concentrated on different ways to interfere with the pathways triggered by TGF-β1 activity. One possibility would be to block signal transduction mediated by TGF-β1 and the feasibility of this approach was demonstrated in rodents where the use of small molecules inhibiting TGF-β1 receptors resulted in decreased severity of lung fibrosis induced by irradiation (26, 109). Another approach to limit the activity of TGF-β1 is to use specific small interfering RNA (siRNA). In a study performed by Lu and co-workers it was demonstrated that in a mouse model the use of TGF-β1-siRNA significantly attenuated the increase in TGF-β1 serum levels after the entire thorax irradiation and that the treatment improved histological signs of inflammation and lung oedema (110). Another possibility considered is the use of a replication-defective adenoviral vector (AdTβ-ExR) that increases the levels of soluble TGF-β type II receptor (111). This approach has been tested in irradiated rats where it was shown that the adenoviral vector reduced TGF-β expression, myofibroblast proliferation, and macrophage infiltration in the lungs (111). Interestingly, there is already a drug – ambroxol – which affects TGF-β1 production. Clinical studies showed that oral treatment with ambroxol from the beginning of radiotherapy significantly attenuated the rise of TGF-β1 concentration in the blood, protected patients from declining lung diffusion capacity, and reduced the incidence of pneumonitis and lung fibrosis (11).

Another therapeutic option and promising approach is the application of pirfenidone, an oral synthetic molecule with antifibrotic, antioxidant and anti-inflammatory effects. Pirfenidone inhibits TGF-β1 (112) and has been successfully used in clinical studies (14, 113–115). Recently the use of this drug in a murine model of radiation-induced pulmonary fibrosis revealed an extended median survival time and decreased accumulation of collagen and fibrosis in lung tissues. Pirfenidone also reduced TGF-β1 levels and phosphorylation of Smad3 under experimental conditions (12). Further, pirfenidone has been efficiently used in combination with sunitinib and radiotherapy in Lewis lung carcinoma (116).

### Regulation of Immune Response

One of main component in the development of lung fibrosis is the disbalance of the immune system. It can be speculated that a shift in the immune response from a pro-fibrotic pathway could attenuate and/or prevent lung fibrosis. There is a promising drug, cytosine-phosphate-guanine oligodeoxyribonucleotides (CpG-ODNs), which stimulates the production of pro-inflammatory cytokines and demonstrated a protective effect on post-radiation lung fibrosis development in a mouse model (117, 118). The attenuation of lung fibrosis was associated with reduction of serum concentrations of TGF-β1 and lower amount of hydroxyproline in the lung tissue (117, 118).

### Preventing Alveolar Epithelial Injury

Earlier studies on the role of the alveolar epithelium in the pathogenesis of radiation-induced fibrosis concluded that the development of pneumonitis and pulmonary fibrosis is caused by the disruption of the balance between various cell populations of the pulmonary parenchyma (119). The myofibroblasts of the alveolar wall were formerly expected as the most active cell during fibrogenesis; however, AET I cells, endothelial cells, and the alveolar macrophages are the primary target of injury (108). Subsequently, products of all these cells, particularly of AET I cells, and signals from disturbed intercellular epithelial adhesion and other factors stimulate the AET II cells to proliferate faster, and to secrete many cytokines in autocrine and paracrine mode (119). This results in accelerated proliferation and differentiation of AET II cells into AET I cells (120). This presumably impaired transdifferentiation process includes an increased presence of an intermediate cell type that is cuboidal or flat and expresses both AET I and AET II cell markers (121). The underlying mechanisms which include apoptosis and senescence of alveolar epithelial cells are poorly understood. AET I cells seem to die rather by necrosis than by apoptosis as shown in ultrastructural studies (122, 123). Knockout of AET I-specific proteins (for example PAI-1, Cav-1, RAGE, P2X7R, ICAM-1) in mice show signs of resistance to lung injury and fibrosis (80, 124–127), thus indicating the importance and involvement of the alveolar epithelial cells, namely the AET I cells in fibrogenesis. Preventing alveolar epithelial injury implicates the maintenance of alveolar barrier function e.g., to stabilize epithelial tightness by proper tight junctions as predominant structures between alveolar epithelial cells (128). Disruption of TJs with subsequent loss of alveolar epithelial integrity plays an important role in the development of pulmonary fibrosis (129). Despite the alveolar epithelial barrier function being more resistant to radiation than that of the pulmonary capillary endothelium, intact alveolar epithelial permeability is of critical importance in keeping the alveolar space relatively free of fluid during acute radiation-induced lung injury (130). Promising tools for restoration of alveolar epithelial barrier function in radiation-induced pulmonary fibrosis, however, are missing.

### New Techniques of Radiotherapy

Radiotherapy techniques have changed significantly over the past few decades due to improvements in engineering and computing. The use of state-of-the art equipment employing the recent advances in radiation oncology has a huge potential to limit the risk of radiation induced pulmonary damage. By the end of the 90s, 3-dimentional conformal radiotherapy (3DCRT) was developed, where 3D imaging data is used before the irradiation to design a minimum number of radiation beams with a predefined fixed shape and uniform dose distribution matching the shape of the tumor mass (131). Later this approach was improved into IMRT (intensity-modulated radiotherapy), which allows variation of dose within each beam and typically uses more beams than 3DCRT which leads to more conformal dose distribution (132). This technique was made possible by the use of computer-controlled multi-leaf collimators and advanced treatment planning algorithms that are capable of creating the desired dose variation inside the radiation field (133). In the past decade this approach was further developed into techniques like VMAT (volumetric modulated arc therapy) where the irradiation is carried out while rotating the irradiator around the patient and the radiation dose is accurately shaped to the tumor while minimizing the dose received by the surrounding tissues (134). Further advances include the stereotactic body radiotherapy (SBRT), where immobilization devices and improved real-time imaging have allowed clinicians to administer high ablative doses to precisely target the tumor. The effectiveness of SBRT arises from the cumulative biologically effective dose that can be achieved while maintaining a sharp dose gradient fall off outside the target, preventing dose to critical structures (135). However, this technique is not free of side effects, including airway toxicity and consequent atelectasis, stenosis/stricture, airway necrosis and/or fistula formation (more prominent in patients with centrally located tumors) (136–138), spontaneous pneumothorax (139, 140), pneumonitis (141), chest wall pain and rib fracture (142, 143). Parallel to the development of these photon-based irradiation techniques, research is also focused on particle therapy, which is based on the use of protons and carbon ions to further reduce the radiation dose received by the healthy tissue. In contrast to photons, particle therapy aims to achieve proper radiation dose concentrated predominantly at a precise depth, which allows additional protection to the normal tissue (144, 145).

## Conclusion

Post-radiation lung fibrosis is a common and currently untreatable adverse event of radiation therapy. To date, much is known about the pathophysiological processes of the development of lung fibrosis and pathways supporting the spread of pathological processes in the lungs. This knowledge led to recognition of the key points of fibrotic reaction and hopefully in the future will result in a development of highly effective therapeutic approaches which could be used to improve the duration and quality of life of patients after radiotherapy while at the same time will not limit the effectiveness of the applied anti-neoplastic therapy.

## Author Contributions

NJ, EK, AM, MK, RR, and PS: drafted the manuscript and approved the final version.

## Conflict of Interest

The authors declare that the research was conducted in the absence of any commercial or financial relationships that could be construed as a potential conflict of interest.
